# Bilateral reaching deficits after unilateral perinatal ischemic stroke: a population-based case-control study

**DOI:** 10.1186/s12984-018-0420-9

**Published:** 2018-08-17

**Authors:** Andrea M. Kuczynski, Adam Kirton, Jennifer A. Semrau, Sean P. Dukelow

**Affiliations:** 1 University of Calgary, Calgary, AB T2N 2T9 Canada; 2 Section of Neurology, Department of Pediatrics, Alberta Children’s Hospital Research Institute, Calgary, AB Canada; 3 Department of Clinical Neurosciences, Foothills Medical Centre, Hotchkiss Brain Institute, 1403 – 29th St. NW, Calgary, AB Canada

**Keywords:** Perinatal stroke, Cerebral palsy, Motor control, Reaching, Robotics

## Abstract

**Background:**

Detailed kinematics of motor impairment of the contralesional (“affected”) and ipsilesional (“unaffected”) limbs in children with hemiparetic cerebral palsy are not well understood. We aimed to 1) quantify the kinematics of reaching in both arms of hemiparetic children with perinatal stroke using a robotic exoskeleton, and 2) assess the correlation of kinematic reaching parameters with clinical motor assessments.

**Methods:**

This prospective, case-control study involved the Alberta Perinatal Stroke Project, a population-based research cohort, and the Foothills Medical Center Stroke Robotics Laboratory in Calgary, Alberta over a four year period. Prospective cases were collected through the Calgary Stroke Program and included term-born children with magnetic resonance imaging confirmed perinatal ischemic stroke and upper extremity deficits. Control participants were recruited from the community. Participants completed a visually guided reaching task in the KINARM robot with each arm separately, with 10 parameters quantifying motor function. Kinematic measures were compared to clinical assessments and stroke type.

**Results:**

Fifty children with perinatal ischemic stroke (28 arterial, mean age: 12.5 ± 3.9 years; 22 venous, mean age: 11.5 ± 3.8 years) and upper extremity deficits were compared to healthy controls (*n* = 147, mean age: 12.7 ± 3.9 years). Perinatal stroke groups demonstrated contralesional motor impairments compared to controls when reaching out (arterial = 10/10, venous = 8/10), and back (arterial = 10/10, venous = 6/10) with largest errors in reaction time, initial direction error, movement length and time. Ipsilesional impairments were also found when reaching out (arterial = 7/10, venous = 1/10) and back (arterial = 6/10). The arterial group performed worse than venous on both contralesional and ipsilesional parameters. Contralesional reaching parameters showed modest correlations with clinical measures in the arterial group.

**Conclusions:**

Robotic assessment of reaching behavior can quantify complex, upper limb dysfunction in children with perinatal ischemic stroke. The ipsilesional, “unaffected” limb is often abnormal and may be a target for therapeutic interventions in stroke-induced hemiparetic cerebral palsy.

## Background

Perinatal stroke is an early vascular brain injury that accounts for most hemiparetic cerebral palsy (HCP) [1]. The most common perinatal stroke types are large arterial ischemic strokes (AIS) in the middle cerebral artery territory and smaller fetal periventricular venous infarctions (PVI) of the subcortical white matter [2]. While both types of lesions can damage the sensory-motor system, children with PVI typically show milder impairments. These differences may be attributed to the purely subcortical nature of the venous infarctions compared to the cortical and subcortical injuries sustained within the middle cerebral artery in children with arterial stroke [3]. Differences in timing of the injury may also influence motor development, as PVI lesions are incurred before 32–34 weeks gestation and most arterial lesions are acquired near term.

Most children incur lifelong developmental deficits after perinatal stroke, with 80% having contralateral hemiparesis [3]. While motor impairments of the contralesional upper limb have been the primary focus in rehabilitation, studies have suggested that the “unaffected” ipsilesional limb also shows deficits in coordination, dexterity, strength, and movement speed [4–9]. Many activities of daily living depend on the input and coordination of both arms, therefore developing a better understanding of upper limb impairments may advance therapies and improve outcomes.

Our understanding of motor system development following perinatal stroke has improved markedly in the past decade [3, 10]. At birth, corticospinal tracts are bilateral, with ipsilateral projections withdrawing in the first years of development, resulting in predominantly contralateral limb control [11, 12]. Early perinatal stroke often results in persistent ipsilateral corticospinal projections from the non-lesioned hemisphere to the stroke-affected limbs [13]. Ipsilateral control is associated with poor clinical outcome and reduced hand function in HCP [8, 13, 14]. Much less is known about the development of the control mechanisms for the ipsilesional limb. Gaining a detailed understanding of the movement kinematics of both upper extremities will serve to advance understanding of development and application of potential therapeutic options in HCP.

Robotic technology has been used to objectively quantify complex, discrete sensorimotor functions in adult stroke [15–17] and sensory impairments in children with stroke [18, 19]. The current study aimed to evaluate motor function in hemiparetic children to: 1) characterize movement of the contralesional and ipsilesional upper limbs with a robotic assessment; 2) assess correlations between kinematic and clinical measures of movement. We hypothesized that motor performance, as measured by a visually guided reaching task, in both upper extremities would be more impaired in AIS compared to PVI, and that kinematic parameters would be moderately correlated with clinical motor assessments.

## Methods

### Participant criteria

This was a prospective, case-controlled study involving the Alberta Perinatal Stroke Project, a population-based research cohort [20], and the Foothills Medical Centre Stroke Robotics Laboratory (Calgary, AB) between September 2013 and August 2016. Inclusion criteria were: 1) age 6–19 years, 2) clinical and MRI confirmation of perinatal stroke (AIS, PVI), 3) symptomatic hemiparesis (Pediatric Stroke Outcome Measure [21] sensorimotor component > 0.5 and Manual Abilities Classification System [22] grades I-IV), 4) gestational age > 36 weeks, 5) visual acuity of at least 20/30, and 6) written informed consent/assent. Exclusion criteria were: 1) multifocal stroke, 2) other neurological disorders not attributable to perinatal stroke, 3) severe hemiparesis (Manual Abilities Classification System [22] grade V, indicating no voluntary contraction in the hemiparetic hand), 4) severe spasticity (Modified Ashworth Scale [23] > 3 in any muscle tested), 5) inability to comply with the study protocol, 6) upper limb surgery, botulinum toxin treatment, constraint or brain stimulation therapy within 6 months of study participation.

Typically developing children (6–19 years of age) without neurological impairment were recruited and completed the same evaluations. Written informed consent was obtained from all participants and their parents/guardians. This study was approved by the institutional University of Calgary Conjoint Health Research Ethics Board (CHREB), ID REB15–0136.

### Robotic reaching assessment

**Fig. 1:**
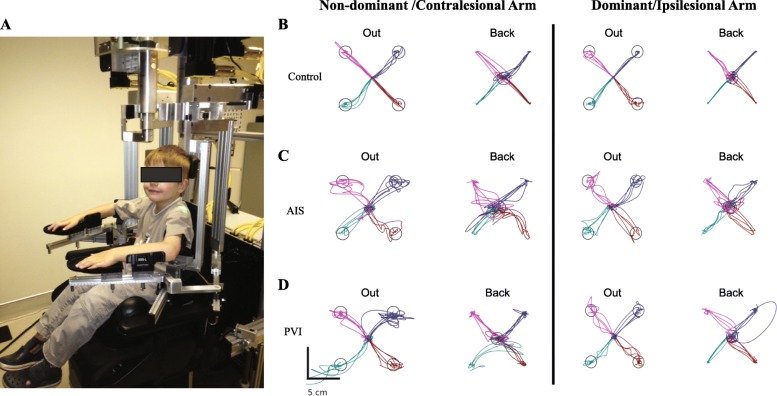
Exemplar reaching performance. **a** 6-year-old control in KINARM robot. Reaching for three participants. **b** A 13-year-old female right-handed control. **c** A 9-year-old female arterial ischemic stroke (AIS) case. The right arm was stroke-affected. **d** An 11-year-old male periventricular venous infarction (PVI) case. The participant’s left arm was stroke-affected

The KINARM robotic exoskeleton (BKIN Technologies Ltd., Kingston, Ontario) quantified movement [24]. Participants were fit to the modified wheelchair base with each arm supported by the exoskeleton (Fig. 1a). The robotic device permits free movement of the participant’s arms in the horizontal plane while monitoring the movement at the shoulder and/or elbow joint [25] and the device has been described in more detail elsewhere [15]. The spatial accuracy of the device in the current task was 0.7 mm with a sampling frequency of 1000 Hz.

A visually guided reaching task evaluated motor performance of both arms. Participants were instructed to move their hand quickly and accurately from a fixed central position to one of four peripheral targets located in the circumference of a circle, separated by 6 cm (Fig. 1). Each participant completed 40 trials with each arm (20 reaches out, 20 back). Peripheral target illumination order was pseudo-randomized. Subjects performed the task once with each arm. All participants performed the task with their dominant limb first. The arms of participants with stroke are herein referred to as either contralesional or ipsilesional, whereas control participants’ arms are either dominant or non-dominant.

### Determination of movement onset/offset in robotic assessments

For each participant, movement onset was calculated during a 500 ms period prior to peripheral target appearance, in which the subject held their hand position in the start target. During this period, two measures were calculated: maximum posture speed (PS_max_) and minimum posture speed (PS_min_). PS_max_ was calculated as the 95th percentile of hand speed in the 500 ms when the hand was positioned in the central target, prior to the illumination of the peripheral target across all trials, while PS_min_ was defined as the 50th percentile of hand speed during this time period [15]. Based on these two thresholds, movement onset was defined as the time when either a) a local minimum in hand speed below PS_max_ was found, or b) the hand speed fell below PS_min_. Movement onset was not recorded if a participant’s hand speed never dropped below PS_max_, or if the participant’s hand left the central target > 2000 ms after the illumination of the peripheral target.

The same thresholds described above were used to define movement offset as the time when the participant reached the peripheral target and a) the first local hand speed minima below PS_max_, or b) hand speed below PS_min_ was found. If a participant did not reach the peripheral target, movement offset was not recorded.

### Description of robotic parameters

Ten parameters, which have been previously described [15], were used to describe different aspects of the reaching movements and calculated for movements reaching out and back:

1. *Postural speed (PS):* hand speed (in cm/s) while holding in the central target. One posture speed score was calculated for each arm.
2. *Reaction time (RT):* time (in seconds) from the peripheral target illumination to the onset of arm movement.
3. *Initial direction error (IDE):* angular deviation (in degrees) between a) a straight line from the hand position at movement onset to the peripheral target, and b) a vector from the hand position at movement onset to the position after the initial movement. The time between movement onset and the first minimum hand speed was defined as the initial stage of movement.
4. *Initial distance ratio (IDR):* the ratio of distance the hand moved during the initial movement to the distance the hand moved between movement onset and offset. A ratio of > 1 represents a distance moved greater than required to reach the peripheral target.
5. *Initial speed ratio (ISR):* the ratio of the maximum hand speed during initial movement to the global hand speed maximum of the trial. In typical reaching movements, healthy participants should move with ISR = 1 as the speed profile of their movements is typically a smooth bell-shaped curve.
6. *Speed maxima count (SMC):* the number of speed peaks associated with arm movement between movement onset and movement offset.
7. *Minimum-maximum speed difference (MMSD):* the difference between speed maxima and minima after the initial movement.
8. *Movement time (MT):* total time (in seconds) from movement onset to offset.
9. *Path length ratio (PLR):* total distance traveled by the hand between movement onset and offset compared to the shortest distance between targets.
10. *Maximum speed (MS):* maximum speed (in cm/s) achieved during the entire movement (40 trials).

Control performance was fit with a line of best fit (SigmaPlot, Systat Software Inc., San Jose) to account for age effects. Ninety-five percent prediction bands for controls were calculated from the mean curves to develop normative ranges. Participants that fell outside these prediction bands for a given parameter were considered to have failed that parameter relative to the control performance.

### Clinical assessments

At the beginning of each session, an experienced therapist performed clinical assessments including:

A. *Muscle strength* of the shoulder, elbow, wrist, and finger was graded using the Medical Research Council scale bilaterally for all participants [26]. Scores ranged from 0 (no muscular contraction) to 5 (normal muscle strength) with a maximum score of 60/arm.
B. *Modified Ashworth Scale (MAS)* assessed tone of shoulders, elbows, and wrists in all children with perinatal stroke [23]. Scores ranged from 0 (no increase in tone) to 4 (rigidity) and were summed to give one total score.
C. *Chedoke-McMaster Stroke Assessment (CMSA)* assessed both contralesional and ipsilesional movement in the arms and hands of children with perinatal stroke [27]. Scores ranged from 0 (paralysis) to 7 (normal movement).
D. *Assisting Hand Assessment (AHA)* assessed 22 real-world activities and measured bimanual upper extremity motor function in hemiparetic children with perinatal stroke [28]. Scores were expressed as logit units, ranging from 0 (no use of the hand) to 100 (normal function).
E. *Melbourne Assessment Unilateral Upper Limb Function (MA)* assessed 16 tasks of reaching and grasping of different sized objects to evaluate finger dexterity and speed of movement in hemiparetic children with perinatal stroke [29]. Scores ranged from 0 (unable to perform) to 100 (no difficulty).
F. *Purdue pegboard test (PPB)* (LaFayette Instrument Co, LaFayette, IN) tested fine motor function of each hand separately in all participants. Participants picked up one peg at a time and successively filled them down a sequence of holes, as quickly as possible in 30 s. This test was repeated twice and the best score used in analysis.
G. *Modified Edinburgh Handedness Inventory* determined hand dominance using a 10-item assessment [30]. Right arm use for an item was scored + 10 while left arm use was scored − 10. Equal use of both limbs (ambidextrous) was scored 0. Completely right-hand dominant individuals scored + 100, while left-hand dominant individuals scored − 100. Subjects scoring between − 50 and + 50 (with the exception of 0) were classified as mixed handedness and were classified according to their self-reported handedness.
H. Visual fields were assessed using confrontation and scored as normal or abnormal (hemianopsia, quadrantanopsia).
I. The Behavioural Inattention Test (BIT) assessed visuospatial function using six conventional subtests (line bisection, line crossing, star cancellation, letter cancellation, figure and shape copying, representational drawing) for a total possible score of 146 with scores < 130 indicating hemispatial neglect [31].

### Statistical analyses

Statistical analyses were performed using SigmaPlot and SPSS (IBM, Armonk, NY). Kolmogorov-Smirnov tests determined the normality of data distributions. A one-way ANCOVA was conducted using SPSS to determine whether differences existed between the three groups (AIS, PVI, controls) for each reaching parameter while controlling for age. Post-hoc pairwise comparisons were then conducted using Bonferroni corrections for multiple comparisons (α = 0.05). Within each group, Mann-Whitney U-tests or paired t-tests compared performance between both upper limbs and between out and back performance of each limb. Partial Spearman’s correlations controlling for age assessed the relationship of bilateral reaching parameters with clinical assessments (controlled for comparisons to 6 clinical measures, α = 0.05, *p* < 0.008). In the case of missing clinical data, participants were removed from the analysis.

## Results

**Fig. 2:**
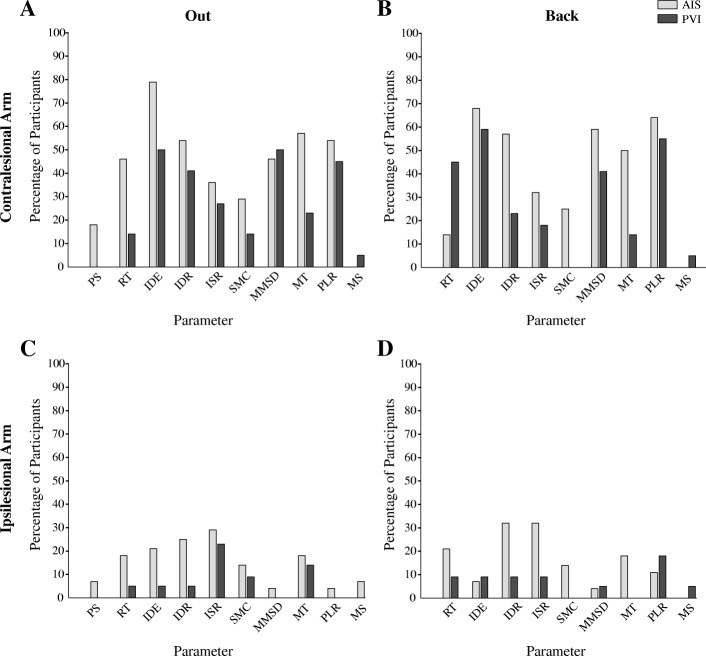
Failure on reaching parameters. The percentage of individuals within the arterial ischemic stroke (AIS) and periventricular venous infarction (PVI) groups that fell outside 95% range of controls for reaches made out (**a**, **c**) and back (**b**, **d**) for each arm. Abbreviations: posture speed (PS), reaction time (RT), initial direction error (IDE), initial distance ratio (IDR), initial speed ratio (ISR), speed maxima count (SMC), minimum-maximum speed difference (MMSD), movement time (MT), path length ratio (PLR), maximum speed (MS)

Table 1 summarizes the demographic and clinical characteristics of the participants. Overall, 28 AIS, 22 PVI, and 147 healthy controls were included in the study. Examples of bilateral reaching performance are shown in Fig. 1. An exemplar control demonstrates normal reaching movements of both upper extremities. Typical AIS and PVI participants demonstrated impairments in performing the same reaching movements. The percentage of AIS and PVI participants that failed each robotic parameter is in Fig. 2. Table 2 describes the mean performance of the three groups across each reaching parameter.

**Table 1:** Demographic information

	AIS		PVI		Controls	
Number of Subjects	28		22		147	
Age (years)	12.5 ± 3.9		11.5 ± 3.8		12.7 ± 3.9	
Sex (female, male)	10, 18		8, 14		71, 76	
Paretic Limb (L, R)	10, 18		11, 11		–	
Handedness (L, R, M)	18, 10, 0		8, 12, 2		8, 127, 12	
Logit AHA [0–100]	61.3 ± 19.9 (32–100)^a^		75.2 ± 16.0 (55–100)^c^		–	
MA [0–100]	69.1 ± 21.3 (31–100)^a^		89.4 ± 10.7 (64–100)^c^		–	
BIT [0–146]	129.5 ± 22.2 (56–145)^b^		138.5 ± 5.6 (122–146)^b^		–	
	Contralesional	Ipsilesional	Contralesional	Ipsilesional	Non-dominant	Dominant
Strength [0–60]	48.2 ± 8.7 (30–60)	59.9 ± 0.4 (58–60)^b^	55.6 ± 3.9 (47–60)	60.0 ± 0.0 (60)^b^	60.0 ± 0.2 (58–60)^d^	60.0 ± 0.09 (59–60)^b^
MAS [0–24]	3.64 ± 3.1 (0–15)	0.0 ± 0.0	1.91 ± 2.6 (0–10)	0.0 ± 0.0	–	–
CMSA Arm [1–7]	[0, 0, 13, 2, 5, 3, 5]	[0, 0, 0, 0, 0, 4, 24]	[0, 0, 3, 1, 4, 4, 10]	[0, 0, 0, 0, 0, 5, 17]	–	–
CMSA Hand [1–7]	[0, 5, 12, 5, 3, 2, 1]	[0, 0, 0, 0, 1, 5, 22]	[0, 1, 0, 10, 10, 1]	[0, 0, 0, 0, 8, 14]	–	–
PPB	1.81 ± 3.4 (0–11)	12.5 ± 2.0 (10–16)	5.50 ± 3.7 (0–11)	13.1 ± 1.7 (10–16)	13.8 ± 2.3 (8–19)^c^	14.9 ± 2.4 (8–21)^b^

Mean ± SD shown, (round brackets contain range). CMSA shown as the number of participants with each score on the 7-point scale. Abbreviations: arterial ischemic stroke (AIS), periventricular venous infarction (PVI), L (left), R (right), M (mixed), Assisting Hand Assessment (AHA), Melbourne Assessment of Unilateral Upper Limb Function (MA), Behavioural Inattention Test (BIT), Modified Ashworth Scale (MAS), Chedoke-McMaster Stroke Assessment (CMSA), Purdue Pegboard (PPB). Missing data from ^a^ eight, ^b^ one, ^c^ nine, and ^d^ three participants

**Table 2:** Comparison of mean robotic visually guided reaching performance between the stroke groups and healthy controls

	AIS		PVI		Controls	
	Out	Back	Out	Back	Out	Back
*Contralesional/non-dominant upper extremity*						
PS (cm/s)	0.29 ± 0.2^*^		0.20 ± 0.09		0.21 ± 0.1	
RT (s)	0.49 ± 0.2^*†^	0.46 ± 0.1^*†^	0.43 ± 0.1^ǂ^	0.42 ± 0.1^ǂ^	0.37 ± 0.08	0.36 ± 0.09
IDE (°)	10.24 ± 6.2^*†^	10.70 ± 8.6^*†^	8.60 ± 4.3^ǂ^	8.30 ± 4.1^ǂ^	4.16 ± 1.4	3.56 ± 1.4
IDR	0.65 ± 0.2^*^	0.69 ± 0.1^*†^	0.69 ± 0.2^ǂ^	0.77 ± 0.1^ǂ^	0.84 ± 0.1	0.89 ± 0.08
ISR	0.92 ± 0.07^*^	0.93 ± 0.08^*†^	0.94 ± 0.06^ǂ^	0.97 ± 0.04	0.98 ± 0.03	0.98 ± 0.03
SMC	2.74 ± 0.7^*^	2.73 ± 0.9^*†^	2.49 ± 0.5^ǂ^	2.24 ± 0.4	2.15 ± 0.4	2.00 ± 0.4
MMSD (cm/s)	2.03 ± 1.6^*^	2.21 ± 1.2^*^	2.11 ± 1.4^ǂ^	1.95 ± 1.5^ǂ^	0.79 ± 0.6	0.80 ± 0.7
MT (s)	1.36 ± 0.3^*†^	1.33 ± 0.3^*†^	1.20 ± 0.3^ǂ^	1.15 ± 0.2^ǂ^	0.97 ± 0.2	0.95 ± 0.2
PLR	1.59 ± 0.5^*^	1.60 ± 0.4^*†^	1.51 ± 0.3^ǂ^	1.45 ± 0.2^ǂ^	1.20 ± 0.1	1.22 ± 0.1
MS (cm/s)	13.21 ± 1.9^*†^	14.00 ± 1.7^*^	16.08 ± 3.7	16.12 ± 3.9	16.80 ± 4.0	17.76 ± 4.3
*Ipsilesional/dominant upper extremity*						
PS (cm/s)	0.23 ± 0.2		0.22 ± 0.1		0.21 ± 0.1	
RT (s)	0.44 ± 0.1^*^	0.42 ± 0.1^*^	0.41 ± 0.1	0.40 ± 0.1	0.35 ± 0.08	0.35 ± 0.08
IDE (°)	6.12 ± 3.3^*†^	5.67 ± 2.1^*^	5.01 ± 1.8	5.08 ± 2.2	4.52 ± 1.7	3.97 ± 2.0
IDR	0.76 ± 0.1^*^	0.81 ± 0.1^*^	0.79 ± 0.1	0.85 ± 0.1	0.84 ± 0.1	0.88 ± 0.08
ISR	0.95 ± 0.04^*^	0.96 ± 0.05^*^	0.96 ± 0.04^ǂ^	0.97 ± 0.04	0.98 ± 0.03	0.99 ± 0.02
SMC	2.47 ± 0.6^*^	2.22 ± 0.5	2.21 ± 0.4	2.03 ± 0.4	2.20 ± 0.4	2.03 ± 0.4
MMSD (cm/s)	0.84 ± 0.6	0.74 ± 0.7	0.89 ± 0.4	0.80 ± 0.9	1.03 ± 0.8	1.08 ± 0.9
MT (s)	1.09 ± 0.2^*^	1.05 ± 0.2^*†^	1.00 ± 0.2	0.94 ± 0.2	0.94 ± 0.2	0.90 ± 0.2
PLR	1.22 ± 0.1	1.25 ± 0.2	1.21 ± 0.1	1.22 ± 0.1	1.22 ± 0.1	1.23 ± 0.1
MS (cm/s)	13.64 ± 2.6^*^	14.32 ± 2.1^*†^	15.91 ± 4.1	17.11 ± 4.6	16.94 ± 3.7	18.09 ± 3.8

Scores are shown as mean ± standard deviation. Statistical significance (p < 0.05) is indicated for differences between AIS and controls (^*^), PVI and controls (^ǂ^), and between AIS and PVI groups (^†^). Abbreviations: arterial ischemic stroke (AIS), periventricular venous infarction (PVI), posture speed (PS), reaction time (RT), initial direction error (IDE), initial distance ratio (IDR), initial speed ratio (ISR), speed maxima count (SMC), minimum-maximum speed difference (MMSD), movement time (MT), path length ratio (PLR), maximum speed (MS)

### Contralesional / non-dominant reaching

**Fig. 3:**
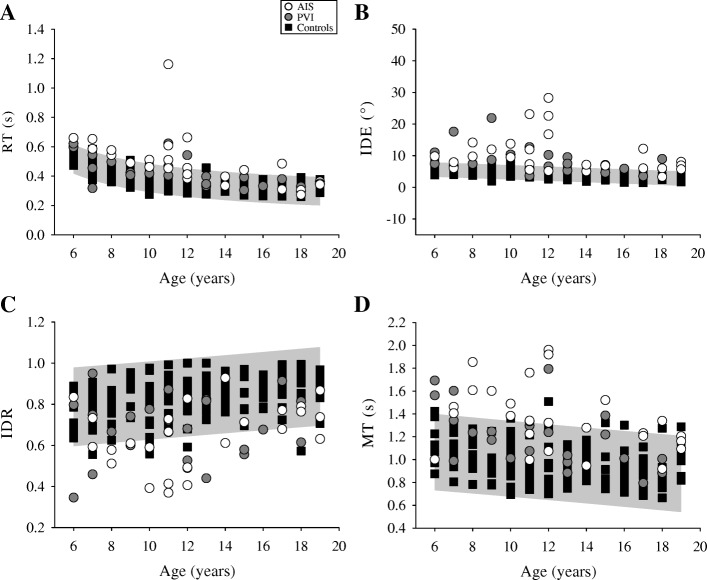
Contralesional/non-dominant reaching performance. The reaching performance out for (**a**) reaction time (RT), (**b**) initial direction error (IDE), (**c**) initial distance ratio (IDR), and (**d**) movement time (MT) is shown for the arterial ischemic stroke (AIS), periventricular venous infarction (PVI), and control subjects represented by open circles, filled circles and black squares, respectively. The 95% prediction bands of control performance (grey box) with their non-dominant limb in each measure are shown

The AIS group showed impairments in all reaching parameters when reaching out with their contralesional arm compared to the non-dominant arm of controls (Table 2). PVI participants were impaired in 8 of 10 parameters. Both stroke groups had slower reaction times (F(2,196) = 28.8, *p* < 0.001), larger initial direction errors (F(2,196) = 67.1, *p* < 0.001), moved with longer initial movements (F(2,196) = 43.5, *p* < 0.001), and had greater movement time (F(2,196) = 51.5, *p* < 0.001; Fig. 3). The AIS group had longer reaction times (*p* < 0.01), larger initial direction error (*p* = 0.05), and longer movement times (*p* < 0.01) with slower maximum speed (*p* < 0.05) compared to PVI when reaching out.

**Fig. 4:**
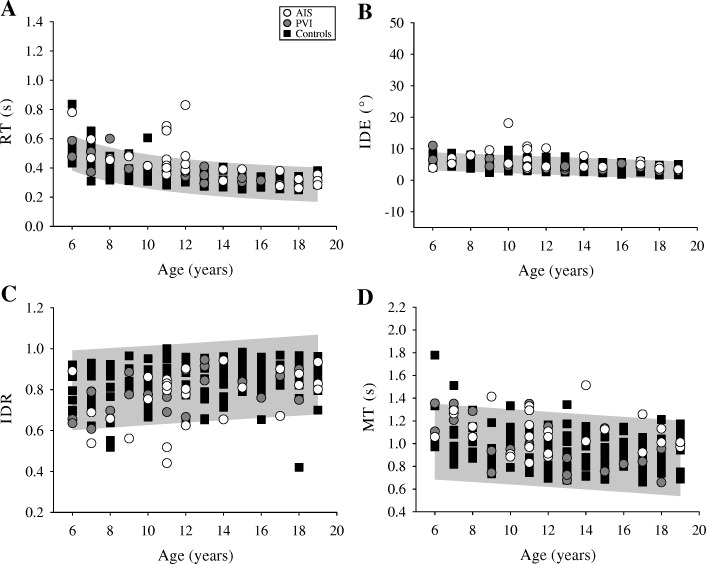
Ipsilesional/dominant reaching performance. The reaching performance out for (**a**) reaction time (RT), (**b**) initial direction error (IDE), (**c**) initial distance ratio (IDR), and (**d**) movement time (MT) is shown for the arterial ischemic stroke (AIS), periventricular venous infarction (PVI), and control subjects represented by open circles, filled circles and black squares, respectively. The 95% prediction bands of control performance (grey box) with their dominant limb in each measure are shown

When reaching back to the central target with their contralesional arm, the AIS group again showed impairments in all 10 reaching parameters. Conversely, the PVI group showed impairments in 6 parameters (Table 2). Both stroke groups had slower reaction times (F(2,196) = 22.8, p < 0.05), larger initial direction errors (F(2,196) = 55.3, *p* < 0.001), and slower movement times (F(2,196) = 49.0, *p* < 0.001; Fig. 4) than controls. The AIS group differed from PVI on 7 parameters. AIS subjects had greater reaction time (*p* < 0.05) and initial direction error (*p* < 0.05), longer (*p* < 0.01) and slower (*p* < 0.001) initial movements, made more sub-movements (*p* = 0.001), and had slower (*p* = 0.001) and longer movements (*p* < 0.01).

### Differences in contralesional / non-dominant reaches out and back

Comparing the reaching out to reaching back, controls had slower reaction times (Z = − 2.78, *p* < 0.01), larger initial direction errors (Z = − 5.92, p < 0.001), shorter initial movements (Z = 5.58, p < 0.001), moved slower initially (Z = 2.34, *p* < 0.05), made more sub-movements (Z = − 5.09, p < 0.001), shorter movements (t(146) = − 2.03, *p* < 0.05), and slower maximum speed (t(146) = − 6.57, *p* < 0.001) when reaching out. The AIS group followed a similar trend with shorter (t(26) = − 2.03, *p* = 0.05), and slower movements (Z = 2.11, *p* < 0.05 and t(26) = − 2.30, p < 0.05, respectively) in reaching out versus back, while the PVI group had shorter (t(21) = − 2.73, *p* = 0.01) and slower speed (t(21) = − 3.59, *p* < 0.01) movements with more sub-movements (t(21) = 2.70, *p* = 0.01) reaching out versus back.

### Ipsilesional / dominant reaching

Reaching out with their ipsilesional arm, the AIS group showed impairments in 7 parameters (Table 2). Compared to controls’ dominant arm reaching, the AIS group had slower reaction times (F(2,196) = 16.2, *p* < 0.001), larger initial direction error (F(2,196) = 9.16, *p* < 0.001), shorter (F(2,196) = 7.69, *p* = 0.001) and slower (F(2,196) = 17.1, *p* < 0.001) initial movements, more sub-movements (F(2,196) = 4.07, *p* < 0.05), greater movement time (F(2,196) = 8.89, *p* < 0.001), and slower speed (F92196) = 9.80, *p* < 0.001). PVI participants only showed reduced initial speed ratio (F(2,196) = 17.1, *p* < 0.01) compared to controls. As a group, AIS participants had greater initial direction error when reaching out compared to PVI (*p* < 0.05).

Reaching back, the AIS group showed impairment in 6 parameters while the PVI group was not different from controls. AIS participants had slower reaction times (F(2,196) = 11.8, *p* < 0.001), greater initial direction error (F(2,196) = 10.0, *p* < 0.001), shorter (F(2,196) = 10.2, *p* < 0.001) and slower (F(2,196) = 13.8, p < 0.001) initial movements, greater movement time (F(2,196) = 8.98, p < 0.001), and slower overall speed (F(2,196) = 12.0, p < 0.001). The AIS group had greater movement time (*p* < 0.05) and slower maximum speed (*p* < 0.05) in their movements compared to the PVI group reaching back with their ipsilesional limb.

### Differences in ipsilesional / dominant reaches out and back

Control participants had slower reaction times (Z = − 2.02, *p* < 0.05), greater initial direction error (Z = − 4.92, *p* < 0.001), shorter initial movements (Z = 5.35, *p* < 0.001), slower initial speed (Z = 2.95, *p* < 0.001), more sub-movements (t(146) = 5.72, *p* < 0.001), shorter movement time (t(146) = 5.27, *p* < 0.001), and slower maximum hand speed (t(146) = − 9.93, *p* < 0.001) when reaching out compared to back. The AIS group made shorter initial movements (t(27) = − 2.12, *p* < 0.05), more sub-movements (t(27) = 3.06, *p* < 0.01), had greater movement time (Z = − 2.14, *p* < 0.05), and lower maximal speed (t(27) = − 2.31, p < 0.05) when reaching out. Similar to AIS, PVI subjects demonstrated shorter initial movements (t(21) = − 3.04, *p* < 0.01), reduced initial speed (Z = 2.02, *p* = 0.05), more sub-movements (t(21) = 2.40, p < 0.05), longer movement times (t(21) = 2.94, p < 0.01), and slower overall speed (t(21) = − 2.96, *p* < 0.01) when reaching out versus back.

### Inter-limb differences

When reaching out, controls displayed faster reaction times (Z = − 4.46, *p* < 0.001) and larger min-max speed differences (t(146) = − 3.82, *p* < 0.001) with the dominant arm, but all other parameters were similar between limbs. The AIS group displayed slower reaction times (Z = − 3.22, *p* = 0.001), larger initial direction error (Z = − 3.58, *p* < 0.001), smaller initial movements (Z = 4.13, *p* < 0.001), larger min-max speed difference (Z = − 3.87, *p* < 0.001), slower movement time (t(26) = 4.13, *p* < 0.001) and longer movements (Z = − 4.20, *p* < 0.001) when reaching with the contralesional arm. The PVI group demonstrated larger initial direction errors (Z = − 3.78, *p* < 0.001), min-max speed differences (t(21) = 3.83, *p* < 0.001), slower movement time (t(21) = 3.26, *p* < 0.01) and longer movements (Z = − 4.11, *p* < 0.001) with the contralesional versus the ipsilesional arm.

Reaching back, controls demonstrated slower reaction time (Z = − 3.08, *p* < 0.01), min-max speed difference (Z = 3.59, p < 0.001), and slower movement time (t(146) = 3.72, p < 0.001) with their non-dominant arm. The AIS group displayed greater reaction time (Z = − 3.17, p < 0.01), larger initial direction error (Z = − 3.39, *p* < 0.001), shorter initial movements (t(26) = − 4.36, *p* < 0.001), greater min-max speed difference (t(26) = 5.73, *p* < 0.001), slower (t(26) = 4.15, p < 0.001) and longer movements (Z = − 4.18, *p* < 0.001) with their contralesional versus their ipsilesional arm. The PVI group showed greater initial direction error (t(21) = 3.60, p < 0.01), larger min-max speed difference (t(21) = 3.35, p < 0.01), slower (t(21) = 4.65, p < 0.001) and longer movements (t(21) = 4.62, p < 0.001) with the contralesional arm.

### Hemispheric damage and reaching performance

AIS participants with left (*n* = 18) versus right (*n* = 10) hemispheric damage showed no statistically significant differences in reaching performance out and back with either arm. Further no statistically significant differences were observed in PVI cases with left (*n* = 11) versus right (n = 11) hemispheric damage when reaching out and back with either limb.

### Clinical assessments and reaching performance

AHA scores were lower in AIS cases (61.3 ± 20.5) compared to the PVI group (75.2 ± 16.7, t(31) = − 2.04, *p* < 0.05; Table 1). MA scores were lower in the AIS (69.1 ± 21.8) group compared to PVI (89.4 ± 11.1, U = 67.0, p < 0.05). Ipsilateral deficits determined by the CMSA (score = 6) were found in four AIS and five PVI participants. CMSA scores were lower in AIS than PVI cases in their contralesional arm (U = 171.5, *p* < 0.01) and hand (U = 82.5, *p* < 0.001) than the ipsilesional. CMSA scores of the ipsilesional arm (U = 282, *p* = 0.45) and hand (U = 266, *p* = 0.30) were comparable between AIS and PVI groups. Several reaching parameters were moderately correlated with our clinical measures in the AIS group (Table 3). While some significant correlations were observed between clinical measures and robotic parameters in the ipsilesional limb of the AIS group, we observed a greater number of significant correlations in the contralesional limb of the AIS group. No statistically significant correlations were observed between clinical and robotic reaching scores out or back with either limb in PVI.

**Table 3:** Clinical correlations with robotic reaching performance

	AIS				PVI			
	Contralesional		Ipsilesional		Contralesional		Ipsilesional	
	Out	Back	Out	Back	Out	Back	Out	Back
*AHA*								
PS (cm/s)	0.070		−0.076		− 0.33		0.00	
RT (s)	−0.32	− 0.19	− 0.39	− 0.41	− 0.36	− 0.23	0.0060	− 0.054
IDE (°)	− 0.58	− 0.57	− 0.20	0.053	− 0.18	− 0.47	− 0.014	− 0.090
IDR	0.51	0.63^*^	0.14	0.065	0.34	0.29	0.21	0.26
ISR	0.35	0.25	−0.12	0.17	0.22	0.26	0.14	0.091
SMC	−0.67^*^	− 0.48	− 0.041	0.028	− 0.33	0.26	0.079	0.35
MMSD (cm/s)	−0.55	− 0.75^*^	− 0.40	− 0.24	− 0.42	0.14	− 0.11	0.43
MT (s)	−0.60^*^	− 0.72^*^	0.24	0.066	−0.64^*^	− 0.55	− 0.19	− 0.062
PLR	− 0.41	− 0.52	− 0.43	− 0.18	− 0.44	− 0.32	0.24	0.36
MS (cm/s)	0.073	0.063	−0.41	− 0.40	0.31	0.47	0.48	0.45
*MA*								
PS (cm/s)	0.10		0.11		−0.46		−0.16	
RT (s)	− 0.36	−0.37	− 0.31	−0.36	− 0.53	−0.36	− 0.42	−0.32
IDE (°)	−0.47	−0.46	− 0.12	−0.022	− 0.25	−0.49	− 0.20	−0.061
IDR	0.32	0.46	0.12	0.13	0.13	0.22	0.17	0.040
ISR	0.31	0.19	−0.039	0.15	0.0050	0.44	−0.11	−0.14
SMC	−0.53	−0.32	− 0.0050	−0.0040	− 0.23	0.35	0.083	0.52
MMSD (cm/s)	−0.42	−0.63^*^	− 0.45	−0.45	− 0.39	0.090	− 0.096	0.49
MT (s)	−0.50	−0.56	0.12	−0.047	− 0.34	−0.34	− 0.18	0.0020
PLR	−0.34	−0.50	− 0.47	−0.26	− 0.37	−0.24	0.15	0.27
MS (cm/s)	0.039	0.059	−0.34	−0.31	0.14	0.27	0.44	0.30
*CMSA Arm*								
PS (cm/s)	0.14		−0.29		−0.18		−0.022	
RT (s)	−0.19	−0.16	− 0.022	−0.070	− 0.38	−0.27	0.19	0.17
IDE (°)	−0.48	−0.52^*^	0.16	−0.28	− 0.39	−0.19	− 0.37	0.0090
IDR	0.56^*^	0.69^*^	−0.062	0.25	0.54	0.22	0.051	−0.32
ISR	0.50	0.35	−0.41	−0.072	0.47	0.34	−0.24	−0.18
SMC	−0.64^*^	−0.48	0.13	−0.081	− 0.29	0.11	0.18	−0.0010
MMSD (cm/s)	−0.49	−0.55^*^	− 0.15	−0.0040	− 0.34	0.11	− 0.17	0.017
MT (s)	−0.61^*^	−0.56^*^	0.19	0.35	−0.33	−0.13	0.26	−0.071
PLR	−0.45	−0.51^*^	0.20	−0.061	− 0.36	−0.16	− 0.037	−0.031
MS (cm/s)	−0.061	0.25	0.010	−0.19	0.16	0.29	−0.025	0.067
*CMSA Hand, mean (SD)*								
PS (cm/s)	−0.11		0.10		0.31		−0.15	
RT (s)	−0.26	−0.37	0.086	0.10	−0.26	−0.47	− 0.039	−0.027
IDE (°)	−0.43	−0.37	0.013	−0.050	− 0.047	0.21	− 0.083	−0.074
IDR	0.36	0.36	0.22	0.14	0.37	−0.010	−0.18	− 0.31
ISR	0.21	0.16	0.045	−0.014	0.23	−0.21	−0.12	− 0.43
SMC	−0.59^*^	− 0.32	−0.26	− 0.20	−0.15	− 0.14	0.36	0.13
MMSD (cm/s)	−0.56^*^	−0.55^*^	− 0.33	−0.15	− 0.0050	0.056	− 0.034	−0.22
MT (s)	−0.65^*^	−0.49	− 0.37	−0.070	− 0.33	−0.48	0.33	0.10
PLR	−0.53^*^	−0.57^*^	− 0.034	−0.096	− 0.11	−0.25	− 0.38	−0.25
MS (cm/s)	−0.13	0.12	0.10	0.0010	0.18	0.15	−0.41	−0.47
*PPB*								
PS (cm/s)	−0.0020		−0.20		0.23		−0.30	
RT (s)	−0.24	−0.27	− 0.51^*^	−0.56^*^	− 0.27	−0.21	− 0.53	−0.52
IDE (°)	−0.37	−0.35	0.061	−0.45	− 0.080	−0.21	− 0.45	−0.15
IDR	0.55^*^	0.57^*^	0.13	0.35	0.52	0.19	0.49	−0.22
ISR	0.30	0.23	−0.075	0.078	0.40	0.12	0.066	−0.026
SMC	−0.56^*^	−0.41	− 0.026	−0.10	− 0.30	0.016	− 0.19	0.27
MMSD (cm/s)	−0.53^*^	−0.55^*^	0.021	−0.45	− 0.32	0.055	− 0.31	0.28
MT (s)	−0.61^*^	−0.54^*^	− 0.022	−0.13	− 0.56^*^	−0.50	− 0.11	−0.13
PLR	−0.40	−0.44	− 0.031	−0.26	− 0.32	−0.29	− 0.20	0.029
MS (cm/s)	0.069	0.16	0.19	−0.040	0.29	0.36	0.27	0.33

Partial Spearman’s correlations controlling for the effects of age were conducted between robotic measures and clinical motor assessments. R values of each correlation are shown. An asterisks (*) denotes significant correlations following Bonferroni correction for multiple comparisons (6 comparisons, α = 0.05, p < 0.008). Abbreviations: arterial ischemic stroke (AIS), periventricular venous infarction (PVI), Assisting Hand Assessment (AHA), Melbourne Assessment of Unilateral Upper Limb Function (MA), Chedoke-McMaster Stroke Assessment (CMSA), Purdue Pegboard (PPB), posture speed (PS), reaction time (RT), initial direction error (IDE), initial distance ratio (IDR), initial speed ratio (ISR), speed maxima count (SMC), minimum-maximum speed difference (MMSD), movement time (MT), path length ratio (PLR), maximum speed (MS)

## Discussion

Children with perinatal stroke demonstrated significant impairments in reaching with their contralesional limb compared to typically developing subjects. On average, deficits were associated with stroke type and were greater in AIS compared to PVI, similar to our recent studies of sensory function [18, 19]. Our findings in the contralesional limb are more detailed than previous kinematic studies in hemiparetic children, but align with observations of increased reaction times and movement times [31–34]. The AIS group also showed significant deficits when completing the reaching task with their ipsilesional limb compared to the dominant arm of controls. Our findings draw attention to the importance of stroke type in determining upper limb motor impairment, and these results can serve to inform models of developmental plasticity following early unilateral brain injury.

Post-stroke therapies often promote task-specific training to improve motor function and independence [33, 34]. In children, modified constraint and bimanual therapies can improve range of movement of the paretic limb as well as motor connectivity in the lesioned hemisphere [35, 36]. To our knowledge, no therapeutic trial has ever targeted specific kinematic deficits. In the present study we examined specific aspects of movement including postural control (e.g. posture speed), movement initiation (e.g. reaction time, initial direction error), feedback/corrective phases of movement (e.g. speed maxima count), as well as overall movement metrics (e.g. movement time). The precision and detail provided by robotic measures may facilitate more individualized therapy approaches in the future specifically targeting difference aspects of movement, either through traditional therapy approaches or more technologically advanced techniques. Further, robots have the ability to measure both small and large changes in motor function over time. These types of measurements have significant potential to quantify responses to novel interventions and also quantify motor development in children. In future research, the types of assessments described in this study could be employed to help determine which patients should be included in clinical trials.

Our confirmation that the “unaffected” limb often has abnormal motor function in children with perinatal stroke and HCP has important implications. Early unilateral brain injuries can result in reduced strength, dexterity, speed, and increased clumsiness of the ipsilesional arm [6, 8, 37–39]. Our study adds detailed kinematic data to this body of literature supporting the concept that a unilateral injury can lead to bilateral motor impairments. Developmental plasticity models suggest sensory function almost always remains contralateral while motor control is often divided between both the lesioned and contralesional hemisphere [40], the degree of which is associated with clinical function [14]. Such dissociation of communication between sensory and motor cortices in the contralateral hemisphere may lead to worse functional deficits. The fact that the better arm for which children with HCP are heavily dependent upon for daily function is specifically impaired in many parameters warrants new focus on improved recognition and possible redirection of therapeutic efforts.

Reaching performance was worse in reaches made out versus back in our groups, regardless of the limb being used. These findings are not surprising and are likely due to the fact that reaches out are to one of four peripheral targets presented pseudo-randomly, but following every peripheral target the reach back was to the same, central target. These findings demonstrate that despite some motor impairment, children with perinatal stroke are almost universally able to use the predictability of a reaching task to optimize their movements.

The two cerebral hemispheres are specialized to facilitate and control different aspects of function. In adults, it has been found that the left hemisphere has greater control over visual integration and initial trajectory features of movement such as movement direction and acceleration, while the right hemisphere is more involved in limb position and posture [41, 42]. Accordingly, left hemispheric damage in adults has been associated with limb apraxia and impaired motor sequencing [43], whereas right hemisphere damage may produce deficits in positional accuracy and target localization [44]. In stark contrast, in our still developing pediatric subjects, we found no differences in reaching performance between stroke cases with left versus right hemisphere damage. An oversimplified, speculative suggestion would be that younger brains harbour “greater” neuroplasticity. More specifically, perinatal stroke studies of language function have shown that side of lesion has little impact on long-term outcome [45]. Our data here supports this and reports new evidence that side of lesion has little impact on major motor function. This is somewhat surprising as the resistance of language to early injury is often attributed to it not being “installed” at the time of injury, whereas the motor system is clearly already functioning before birth.

We also examined how kinematic parameters aligned with traditional clinical measures of motor function. In prior studies in adults with stroke we have demonstrated strong relationships with a number of clinical measures of impairment and disability using similar kinematic measures in both the contralesional [15–17] and ipsilesional limbs [46]. The reaching performance of the contralesional arm showed moderate correlations for the AIS group with multiple different clinical scores. This was not surprising given the importance of reaching in many of the activities required for the performance of these tasks. However, the same associations were not seen in the PVI stroke group, consistent with some of our other data describing clear differences between stroke groups [18, 19].

Our findings of greater impairments in the AIS group are perhaps not surprising and appear consistent with our previous work [18, 19]. It has been hypothesized that children with AIS experience poorer sensorimotor outcomes due to the larger nature of the arterial stroke in the middle cerebral artery territory, affecting both cortical and subcortical structures crucial for both sensory and motor functions [47]. Conversely, the smaller, subcortical PVI lesions selectively injure only the white matter with sparing of the cortex. Additional evidence suggests that sensory pathways can often re-route around such lesions to maintain connections to the appropriate sensory cortex which itself is often damaged in arterial lesions [48]. The timing of these strokes may also have implications for the development of motor and sensory systems where the timing of PVI earlier in gestation when sensory-motor tracts are less developed may facilitate more adaptive developmental plasticity to achieve closer to normal organization and greater function [2, 49]. These models are increasingly informed by both human and preclinical studies [14, 49–51] but precisely how they dictate clinical function remains incompletely understood.

### Limitations

With our large sample of typically developing controls, we were able to assess trends of motor function across childhood. The longitudinal profile of motor control and how it improves across childhood is well described, as are historical reports of gross motor development in children with HCP [52]. However, the kinematics we recorded provide a deeper, more detailed description of how motor control changes with age. Establishing these profiles in healthy controls provides opportunity and context to better interpret dysfunction in children with HCP and perinatal stroke. Despite our large age range, our reported age effects by reaching parameter are cross-sectional rather than developmental. Future longitudinal studies over this age range are required to characterize the developmental effects of perinatal stroke on motor control. Furthermore, additional aspects of upper limb movement, including range of motion or force exertion are not assessed by the current visually guided reaching task and warrant further investigation in future studies.

## Conclusions

In this study, we quantified bilateral motor control in children with perinatal stroke. Traditional interventional strategies focus on improving motor function in the contralesional, paretic arm and independence. We found that several hemiparetic children showed significant impairments in reaching movements of both their contralesional and ipsilesional arms. Although ipsilesional motor deficits may occur less severely than those in the contralesional limb, impairment in the ipsilesional limb may be detrimental especially in stroke survivors that rely on that arm as their primary or preferred limb in daily activities. More research is needed to determine whether treating these ipsilesional impairments could, in fact, improve overall function of the children. Future studies using robotic technology must investigate the clinical relevance of robotically measured motor dysfunction in the upper limbs of children with perinatal stroke.

## Abbreviations

AHA: Assisting Hand Assessment
AIS: Arterial ischemic stroke
BIT: Behavioural Inattention Test
CMSA: Chedoke-McMaster Stroke Assessment
HCP: Hemiparetic cerebral palsy
IDE: Initial direction error
IDR: Initial distance ratio
ISR: Initial speed ratio
KINARM: Kinesiological Instrument for Normal and Altered Reaching Movements
MA: Melbourne Assessment of Unilateral Upper Limb Function
MAS: Modified Ashworth Scale
MMSD: Minimum-maximum speed difference
MS: Maximum speed
MT: Movement time
PLR: Path length ratio
PPB: Purdue pegboard
PS: Posture speed
PS_max_: Maximum posture speed
PS_min_: Minimum posture speed
PVI: Periventricular venous infarction
RT: Reaction time
SMC: Speed maxima count
